# Genomic Analysis Suggests that mRNA Destabilization by the Microprocessor Is Specialized for the Auto-Regulation of Dgcr8

**DOI:** 10.1371/journal.pone.0006971

**Published:** 2009-09-11

**Authors:** Archana Shenoy, Robert Blelloch

**Affiliations:** The Eli and Edythe Broad Center of Regeneration Medicine and Stem Cell Research, Center for Reproductive Sciences, and Department of Urology, University of California San Francisco, San Francisco, California, United States of America; Victor Chang Cardiac Research Institute (VCCRI), Australia

## Abstract

**Background:**

The Microprocessor, containing the RNA binding protein Dgcr8 and RNase III enzyme Drosha, is responsible for processing primary microRNAs to precursor microRNAs. The Microprocessor regulates its own levels by cleaving hairpins in the 5′UTR and coding region of the Dgcr8 mRNA, thereby destabilizing the mature transcript.

**Methodology/Principal Findings:**

To determine whether the Microprocessor has a broader role in directly regulating other coding mRNA levels, we integrated results from expression profiling and ultra high-throughput deep sequencing of small RNAs. Expression analysis of mRNAs in wild-type, Dgcr8 knockout, and Dicer knockout mouse embryonic stem (ES) cells uncovered mRNAs that were specifically upregulated in the Dgcr8 null background. A number of these transcripts had evolutionarily conserved predicted hairpin targets for the Microprocessor. However, analysis of deep sequencing data of 18 to 200nt small RNAs in mouse ES, HeLa, and HepG2 indicates that exonic sequence reads that map in a pattern consistent with Microprocessor activity are unique to Dgcr8.

**Conclusion/Significance:**

We conclude that the Microprocessor's role in directly destabilizing coding mRNAs is likely specifically targeted to Dgcr8 itself, suggesting a specialized cellular mechanism for gene auto-regulation.

## Introduction

MicroRNA maturation involves two processing steps [1]. First, a long primary miRNA (pri-miRNA) is cleaved by the Microprocessor, containing the RNA binding protein Dgcr8 and the RNAseIII enzyme Drosha, to produce a 60–75 nucleotide hairpin precursor miRNA (pre-miRNA) in the nucleus [2], [3], [4], [5], [6]. The pre-miRNA is translocated to the cytoplasm where it is cleaved to a miRNA duplex (∼19–25 nt in length) by the RNAseIII enzyme Dicer [7]. A single strand of the duplex enters the RNA induced silencing complex (RISC) with the help of another RNA binding protein, TRBP [8], [9]. Dicer has roles outside of the maturation of canonical miRNAs. For example, in mouse ES cells, Dicer processes other subclasses of miRNAs including mirtrons and short hairpin RNAs as well as endogenous siRNAs [10]. Similarly, Dicer processes endogenous siRNAs in mouse oocytes [11], [12]. Consistent with these additional roles of Dicer, Dgcr8 knockout (KO) ES cells have less severe phenotypes than Dicer knockout ES cells [13].

The Microprocessor was recently shown to have an additional role in directly destabilizing a mRNA target. Specifically, it can cleave hairpins in the 5′UTR and coding region of the Dgcr8 mRNA, which in turns destabilizes the mature transcript [14], [15], [16]. This negative feedback loop on Dgcr8 suggests the importance of tight homeostatic control of the Microprocessor in normal cellular function. The finding that the Microprocessor can directly influence *Dgcr8* mRNA levels raises the possibility that this mechanism may affect many other mRNAs.

To further test whether there is a broader role of the Microprocessor in the direct regulation of mRNAs, we evaluated the mRNA and small non-coding RNA profiles of wild-type, Dgcr8 KO and Dicer KO cells as well as a recently published data set of small RNAs less than 200 nucleotides from human Hela and HepG2 cell lines [17]. While many mRNAs were differentially expressed between Dgcr8 and Dicer KO ES cells, there was no evidence for Microprocessor based processing of these mRNAs, with the striking exception of Dgcr8 itself. Similarly, analysis of the Hela and HepG2 data sets identified many sequence reads from the Dgcr8 hairpins showing a pattern consistent with Microprocessor activity, but none from any other predicted hairpins within spliced mRNAs. These findings suggest that the Microprocessor's role in directly regulating mRNA levels is specific to auto-regulation of Dgcr8, highlighting the importance of this negative feedback regulation of Microprocessor levels.

## Results

**Figure 1:**
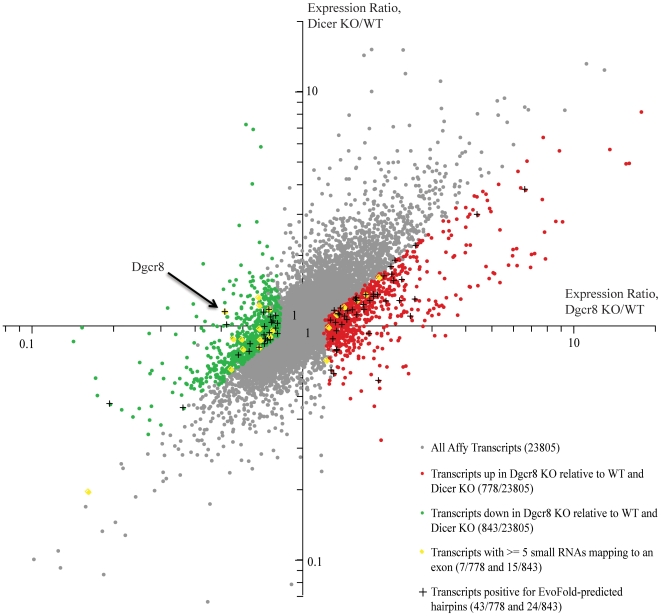
Transcripts differentially regulated in Dgcr8 KO relative to WT and Dicer KO ES cells. The sets of genes differentially up- and down- regulated in Dgcr8 KO relative to Dicer KO and WT ES cells were determined based on a cutoff of FDR <5%. Data are represented as a mean of 3 biological replicates of WT, Dgcr8 KO and Dicer KO arrays. Transcripts positive for EvoFold hairpin predictions and transcripts with 5 or more small RNAs mapping to their exons are shown (see legend). Arrow points to Dgcr8 expression levels, which, as expected, is down in Dgcr8 KO (exon 3 deletion results in premature termination codon and, hence, non-sense mediated RNA decay [33].

mRNAs regulated by a direct Microprocessor cleavage mechanism should be upregulated in cells deficient for the Microprocessor, but not in Dicer deficient cells. Therefore, we evaluated coding mRNA profiling data from wild-type, Dgcr8 KO and Dicer KO mouse ES cells. Normalized mRNA levels in Dgcr8 KO and Dicer KO cells were compared to wild-type ES cells (**Figure 1**). Most mRNAs that were upregulated or downregulated were similarly altered in both mutants. However, similar to previous studies [14], [18], we found multiple mRNAs whose expression were specifically altered in cells that lacked Dgcr8. Using a false discovery rate of 5%, there were 778 transcripts there were upregulated in Dgcr8 KO cells relative to both wild-type and Dicer KO. There were 843 transcripts that were downregulated.

If genes specifically upregulated in Dgcr8 KO cells are normally cleaved by the Microprocessor, there should be hairpin substrates for the complex within these mRNAs. Therefore, we searched for evolutionary conserved hairpins within these mRNAs using predictions generated by the EvoFold algorithm [16]. The 5′UTR hairpin in Dgcr8 was first identified by this method. EvoFold predictions are grouped based on their location in CDS, 5′UTR, 3′UTR, intron and intergenic regions. We determined mouse genome coordinates for EvoFold hairpins in CDS, 5′UTR and 3′UTR regions (see Methods), mapped them to the coding mRNA database, and compared the relative expression levels of all positive hits in Dgcr8 KO, Dicer KO, and wild-type ES cells (**Figure 1**). A total of 824 out of 23805 (3.5%) coding mRNAs contained predicted hairpins. Of these 824, 43 mRNAs were specifically upregulated in Dgcr8 KO cells, while 24 mRNAs were specifically downregulated in the Dgcr8 KO cells. Therefore, there was a subset of genes specifically upregulated in Dgcr8 KO cells that contain predicted hairpins and hence could be direct targets of the Microprocessor.

**Figure 2:**
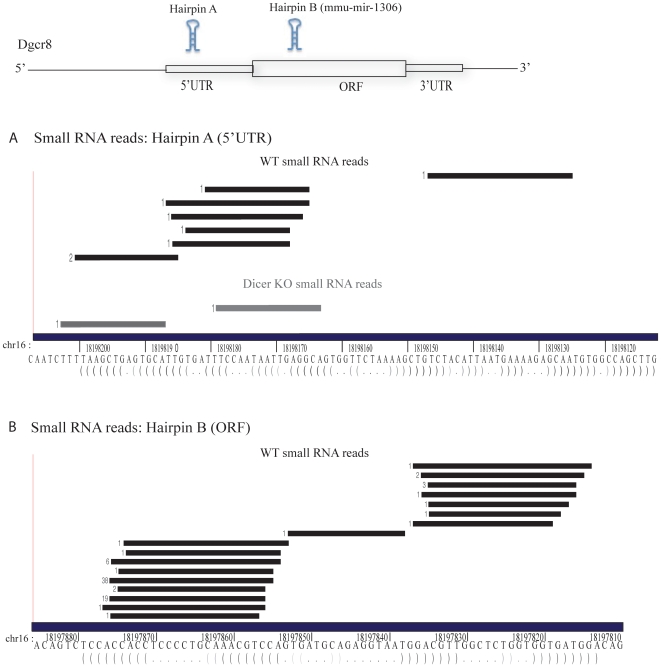
The distribution of reads across hairpins in the first exon of Dgcr8 in mES cells. The location of each small RNA read relative to the exon is represented by a grey bar and was generated using the custom tracks feature on the UCSC genome browser. For each RNA species, the number of reads that were obtained with that sequence is indicated at the left. The predicted secondary structure is represented below the genomic sequence. Genomic coordinates are based on UCSC Known Genes annotations (mm8) (A) 5′UTR hairpin (B) CDS hairpin. Small RNA reads in WT cells are represented by black bars and small RNA reads in Dicer KO cells are represented by a grey bar.

If hairpins within the Dgcr8 KO- upregulated gene set are indeed cleaved by the Microprocessor, we hypothesized that there would be Dgcr8-dependent small RNAs that map to these hairpins. Therefore, we evaluated ultra-high throughput deep sequencing data representing small RNAs ranging from 18-32 nucleotides from the wild-type, Dgcr8 KO and Dicer KO ES cells. As expected, multiple sequence reads mapped to the EvoFold predicted 5′UTR and coding region hairpins of Dgcr8 mRNA in WT cells **(** **Figure 2** **)**. None of the reads mapping to the coding region hairpin were found in either Dgcr8 or Dicer KO libraries confirming their Dgcr8- and Dicer-dependence (**Figure 2B**). Interestingly, two sequence reads mapping to the 5′UTR hairpin were found in the Dicer KO library (**Figure 2A**). One of these reads mapped just 5′ to the hairpin. Such Dgcr8-dependent, Dicer-independent reads have been previously observed at miRNA loci in Drosophila and mouse small RNA sequencing studies and appear to be a 5′ remnant of Drosha cleavage that is further degraded by an unknown 5′-3′ exonuclease [10], [19]. The remaining read that was uncovered in the Dicer KO library had a 5′ end that did not map to the 5′ or 3′ end of the hairpin suggesting that it was a degradation product of the full length hairpin. Analysis of all EvoFold-predicted hairpins in the Dgcr8 KO-upregulated set of coding mRNAs failed to identify a single other hairpin with corresponding small RNAs.

**Figure 3:**
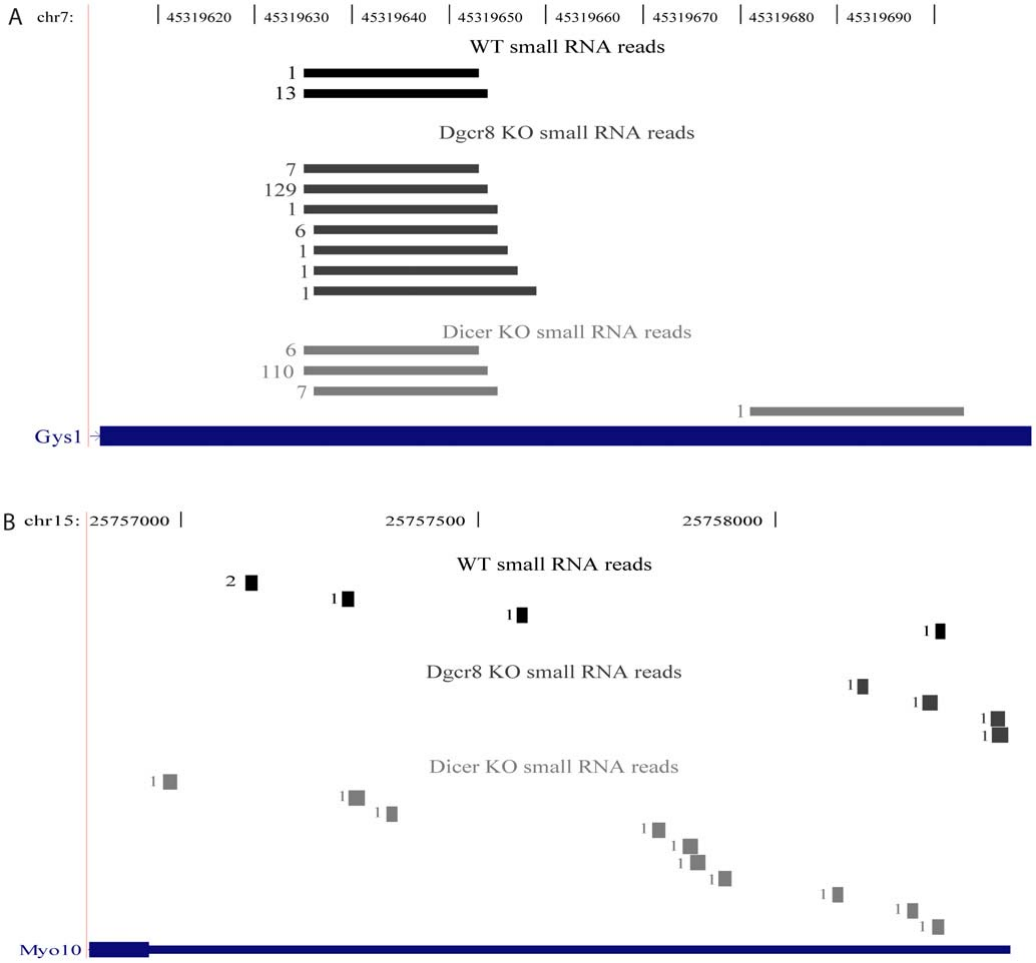
Representative examples of read distribution in exons with >5 reads in WT cells. The location of unique small RNA reads from WT (black bars), Dgcr8 KO (dark grey bars) and Dicer KO (grey bars) are represented. For each RNA species, the number of reads that were obtained with that sequence is indicated at the left. Genomic coordinates are based on UCSC Known Genes annotations (mm8) (A) Example showing reads that are localized to a small window consistent with Microprocessor cleavage but are not Dgcr8-dependent (B) Reads are distributed across the range of the exon and most likely represent degradation products.

Analysis of only EvoFold predicted loci could miss poorly conserved hairpins. Therefore, to extend the analysis, sequencing reads from WT ES cells were mapped to all exons of the transcripts whose expression was altered in Dgcr8 KO versus WT and Dicer KO cells. 7 out of the 778 Dgcr8 KO- upregulated transcripts and 15 out of the 844 downregulated transcripts had at least 5 small RNA reads that overlapped with their exons (**Figure 1**). As Microprocessor activity is predicted to destabilize the mRNAs, we looked more closely at the 7 transcripts upregulated in Dgcr8 KO cells. The small RNAs that mapped within exonic regions of these annotated transcripts fell into two groups based on their distribution. Three had multiple small RNAs with a similar 5′ or 3′ end, consistent with specific endonuclease cleavage **(** **Figure 3A** **and Figure S1, S2)**. The remaining five (two from the same transcript, Arrdc-3) had small RNAs mapping across the exon without shared 5′ or 3′ ends consistent with degradation **(** **Figure 3B** **and Figure S3, S4, S5, S6)**. All of these small RNAs were present in the Dgcr8 null background **(** **Figure 3A–B** **and Figure S1–S6)**. Hence, they are not products of Microprocessor cleavage.

A small number of annotated miRNAs map to exonic regions of coding genes (∼37 in mice) [20]. Therefore, analogous to Dgcr8, the host genes for these miRNAs might be expected to be downregulated by Microprocessor-induced cleavage. Upon examination of the exonic miRNAs, we found only 10 to fully lie within annotated exons **(Table S1)**. We were able to find small RNA reads to three of these exonic miRNAs (mmu-miR-21, mmu-miR-671, mmu-miR-147). However, the mRNA levels of the host genes of these three miRNAs were not altered in the Dgcr8 and Dicer KO ES cells. Therefore, production of these miRNAs does not appear to influence the overall levels of the annotated host mRNAs. Together, these detailed analyses of both mRNA expression profiling and small RNA sequencing data from ES cells failed to uncover any genes other than Dgcr8 that are directly destabilized by the Microprocessor.

**Figure 4:**
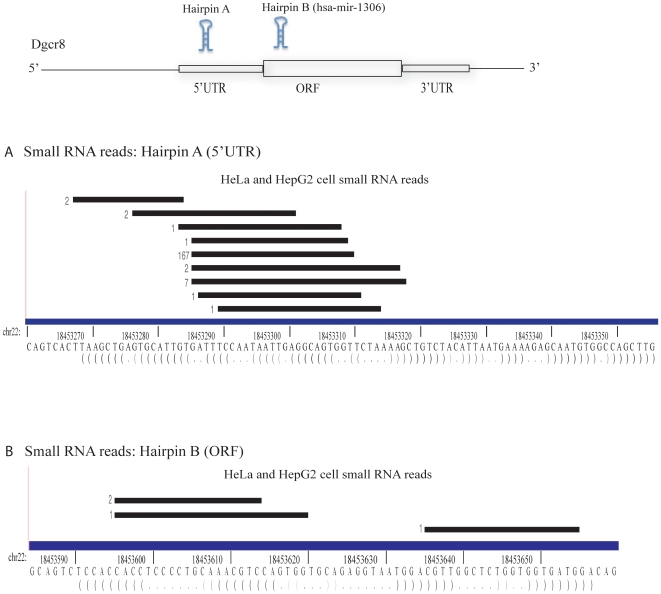
Read distribution across hairpins in the first exon of Dgcr8 in <200nt small RNA sequencing data from HeLa and HepG2 cells. Small RNA locations are presented as in Figure 2. Genomic coordinates are based on UCSC Known Genes annotations (hg18) (A) 5′UTR hairpin (B) CDS hairpin.

It is possible that 18–32 nucleotide small RNA sequencing missed Microprocessor-cleaved exonic hairpins that are sequestered and/or are not processed by Dicer. Microprocessor miRNAs are typically 60–75 nucleotides in length. Therefore, to directly identify these hairpins, we analyzed ultra high-throughput sequencing data sequence sets produced from small RNAs less than 200 nucleotides in length from Hela and HepG2 cells [17]. The forty small RNA libraries generated in the study were derived from whole cell, cytoplasmic and nuclear fractions, as well as from cells following enzymatic treatments that enrich for either mono-, di-, tri-phosphate modified or 5′ capped RNAs. Sequence reads from all forty libraries were mapped to exonic EvoFold hairpins. The largest number of hits, 184, mapped to the Dgcr8 5′UTR hairpin and 4 mapped to the coding region hairpin (**Figure 4**). Most of these reads had a uniform 5′ end consistent with Microprocessor cleavage. There was an additional read just 5′ to the hairpin, a likely remnant of the Microprocessor cleavage, similar to that seen in the ES cell small RNA libraries (**Figure 2**). A large number (166 out of 184) of the 5′ UTR reads were derived from nuclear libraries, consistent with previous work showing that the cleaved 5′UTR hairpin is confined to the nuclear fraction [14]. When mapping reads from the libraries to known pre-miRNA hairpins, many reads extend beyond the known mature miRNA into the loop region of the hairpin **(Figure S7)**, thereby confirming that these libraries contain hairpin products of the Microprocessor cleavage. These findings show that the analysis of the Hela and HepG2 small RNA data sets should identify other hairpins that are cleaved by the Microprocessor even if they are not further processed.

**Figure 5:**
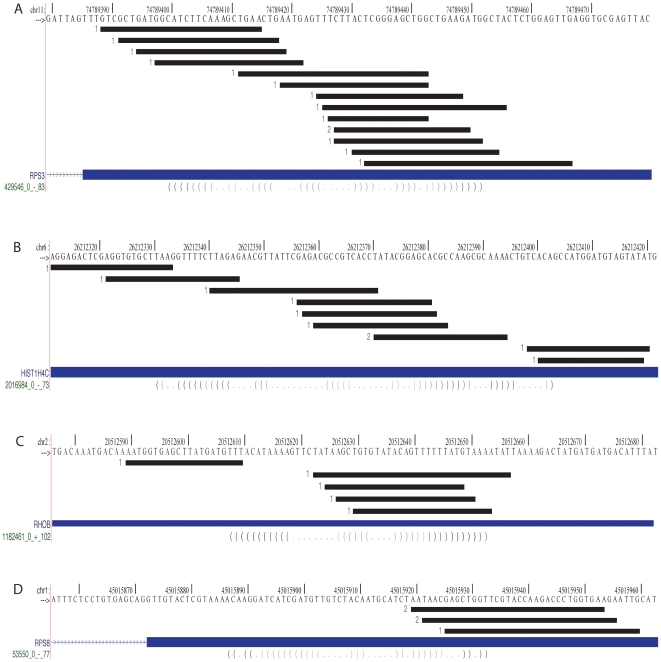
Read distribution across hairpins positive for >5 small RNA reads in HeLa cell <200 nt small RNA sequencing data. Small RNA locations are presented as in Figure 2. Genomic coordinates are based on UCSC Known Genes annotations (hg18) Reads across hairpins in (A) RPS3 (B) HIST1H4C (C) RHOB and (D) RPS8.

In order to identify any other potential mRNA substrates, we next mapped the HeLa and HepG2 datasets to all UTR and CDS EvoFold loci. There were 106 additional EvoFold hairpins containing overlapping small RNAs, although the number of reads mapping to any one of these hairpins was much less than seen for Dgcr8 **(Table S2)**. Only four of these hairpins had at least 5 sequence reads. Furthermore, none of the small RNA reads in these hairpins mapped in a manner consistent with Microprocessor cleavage. That is, they had heterogeneous 5′ and 3′ends and/or the ends went beyond the extremes of the hairpins (**Figure 5A–D**). For example, the second highest-ranking hairpin, which mapped to the gene RPS3, had 14 reads. However, unlike the reads mapping to the Dgcr8 hairpins, they did not have a defined 5′ end, but instead mapped across the locus, more consistent with RNA degradation than Microprocessor cleavage. Therefore, analysis of small RNAs less than 200 nucleotides failed to identify any Evofold loci within exons other than Dgcr8 that are cleaved in a Microprocessor-like fashion.

Again, limiting the analysis to Evofold predicted hairpins would miss non-conserved hairpins. Therefore, we mapped small RNAs from HeLa and HepG2 libraries to exons of transcripts upregulated over 2-fold with siRNA-mediated knockdown of both Drosha and Dgcr8 relative to siGFP. Expression information was extracted from recently published microarray data in HeLa cells (see Methods) [14]. As expected, Dgcr8, which was upregulated in the Drosha knockdown sample, had 188 small RNAs mapping to the first exon. Upon examining protein-coding genes upregulated in both Drosha and Dgcr8 knockdown samples, 31 transcripts had > = 10 small RNAs mapping to at least one exon (45 exons total**, Table S3**). Notably, 15 out of the 31 were genes that encode ribosomal protein subunits, which are highly abundant in cells. Out of the 31, 11 transcripts had small RNA reads distributed over the exon, as would be expected for degradation products. The remaining 20 transcripts had small RNA reads clustering in small window(s) within exons. However, further examination of the regions in these 20 transcripts using RNAfold did not reveal the presence of any good hairpin structures, in contrast to the Dgcr8 small RNA mapping-regions. In summary, analysis of the ultra high-throughput sequence reads of RNAs less than 200 nucleotides, like the ES cell small RNA dataset, showed that a role of the Microprocessor in direct mRNA regulation is likely limited to Dgcr8.

## Discussion

Our findings show a focused role for the Microprocessor in destabilizing coding mRNAs by the direct cleavage and destabilization of spliced transcripts. Indeed, we only find evidence for the cleavage and destabilizaton of Dgcr8. Similar to previous reports, our mRNA profiling analysis of wild-type, Dgcr8, and Dicer deficient cells identifies many mRNAs that are specifically upregulated with the loss of Dgcr8 [18]. The presence of such mRNAs would be consistent with Microprocessor regulation of coding mRNAs through direct cleavage and may be a broadly used mechanism of mRNA regulation. However, closer analysis of these mRNAs and evaluation of ultra-high throughput deep sequencing for small RNAs either in the 18–32 or <200 nucleotide range failed to identify any additional mRNAs that are regulated by such a mechanism. We cannot exclude the possibility that rare examples of Microprocessor-mediated destabilization of mRNAs may be found in specific cellular contexts or at levels too low to be identified using current deep sequencing technology. However, in this study, we examined data from cell lines representing three different tissues: ES (inner cell mass of the blastocyst), Hela (kidney), and HepG2 (liver). Furthermore, in all deep sequencing datasets examined, we find numerous reads to the Dgcr8 hairpins but are unable to find a single additional similar candidate, suggesting that any additional examples would be extremely rare.

The absence of Dgcr8-dependent upregulation of the host transcripts carrying annotated exonic miRNAs is worth noting (**Table S1)**. A number of these annotated exonic miRNAs were not present in our small RNA libraries even though the host gene is clearly expressed. This finding may be the result of mis-annotation of these sequences as miRNAs or that processing of the hairpins is somehow suppressed in ES cells. One example of an annotated exonic miRNA that is present in large numbers in ES cells is miR-21. Its host gene, Tmem49, is not upregulated in Dgcr8 or Dicer knockout ES cells. Possible explanations include: 1) there are alternative transcripts responsible for miR-21 production either from an alternative promoter or an alternative splicing event or 2) only a small subset of the Tmem49 transcripts is processed by the Microprocessor to produce the pre-miR-21 hairpin.

A very specific role for the Microprocessor in destabilizing Dgcr8 and hence providing a negative feedback on Microprocessor levels itself suggests that homeostatic control of microRNA processing is central to normal cellular physiology. This is consistent with recent findings showing that much regulation is occurring at the level of Microprocessor activity. For example hnRNAP, Lin28, and KSRP have been suggested to regulate Microprocessor activity on specific miRNAs [21], [22], [23], [24]. Furthermore, SMAD signaling alters the processing of pri- to pre-miR-21 [25]. A carefully controlled balance between the levels of the Microprocessor and these regulators are likely important for proper physiologic function.

Dgcr8 levels are differentially regulated during development and in cancers. Interestingly, some cancers have decreased, while other cancers have increased levels of Dgcr8 [26], [27]. Similarly, Dicer levels and/or activity appear to be altered in cancers [26], [27], [28], [29]. A direct role for changes in processing activity in cancer is supported by a mouse model of lung cancer where heterozygous loss of Dicer promotes tumor progression [30]. Together, these findings suggest that the biogenesis of miRNAs is not simply a passive process, but rather a tightly controlled one. Therefore, it will be important to determine in greater detail how the level and the activity of the biogenesis machinery influence the molecular constitution of cells.

## Materials and Methods

Solexa sequencing data for Dgcr8 KO, Dicer KO and WT cells were previously published[10]. Information about exonic miRNAs and host genes was extracted from the CoGemir database [20].

### Microarray analysis

Microarray experiments on the wild-type, Dgcr8 KO, and Dicer KO cells were performed by the Gladstone Genomic Core Facility using the Affymetrix 1.0 mouse gene ST arrays with 3 biological replicates per genotype (wild-type (v6.5), *Dgcr8* knockout, *Dicer* knockout ES cells) [10], [31]. Dgcr8 and Dicer knockout ES cell derivation and culture has been previously described [10], [13]. Protocol used for preparation of RNA and hybridization for microarray has been previously described [31]. Array data was normalized using the robust multi-array average (RMA) algorithm. Normalized data has been deposited at GEO (#GSE16923). Genes upregulated and downregulated in Dgcr8 KO relative to WT and Dicer KO were determined by FDR analysis using the SAM software package from Stanford. (http://www-stat.stanford.edu/~tibs/SAM/). Specifically, two sets of genes were determined: 1) Genes upregulated in Dgcr8 KO relative to Dicer KO and 2) Genes upregulated in Dgcr8 KO relative to wild-type. Overlapping transcripts between these two sets of genes were assigned to the group upregulated in Dgcr8 KO relative to both Dicer KO and WT. Genes downregulated in Dgcr8 KO relative to Dicer and WT were determined using the same approach. For the analysis of overlap between small RNAs or predicted hairpins and protein coding mRNAs, we excluded Affy transcripts annotated only as miRNAs, transcripts mapping to the mitochondrial genome, chromosome Y and transcripts missing gene ID annotations.

HeLa cell microarray data was previously published [14]. siGFP, siDrosha and siDgcr8 expression data was averaged for 24 and 48 hr timepoints for each Affy ID, which resulted in 4 biological samples/gene. AffyIDs upregulated at least 2 fold (n = 1195) in both siDrosha and siDgcr8 relative to siGFP were analyzed further.

### Mapping small RNA reads to exons

Small RNA reads from the Solexa sequencing dataset were first mapped to the genome (mouse, version mm8) using Eland. Uniquely mapping small RNA reads were mapped to exons by examining overlap between genomic coordinates of a small RNA read and each exon. Any small RNA overlapping with beginning and end of an exon as well as lying within in an exon was included as a positive hit. Exon information was determined using annotations from the UCSC Known Genes and Ensemble databases (mouse, version mm8) and all transcripts were collapsed to match to Affy ID annotations[32].

For analysis of data from HeLa and HepG2 cells, small RNAs from all libraries were first mapped to the genome (hg18) using Eland. Sequence length of HeLa cell libraries ranged from 15 to 26 nt. Sequence length of HepG2 cell libraries ranged from 15 to 36 nt. Genomic coordinates of the small RNAs were then mapped to exons of transcripts upregulated with siDrosha and siDgcr8 relative to siGFP. Exon information was determined using RefSeq annotations, which were matched to Affy IDs. The positive hits were further filtered manually of snoRNAs. The remaining exons were ranked based on the number of small RNA reads and exons containing >10 small RNAs were analyzed further using custom tracks at the UCSC genome browser. For exons with small RNA reads localized to a small window, sequences surrounding the small RNA reads were extracted and fold predictions were generated using RNAfold.

### Mapping Small RNA reads to miRNA hairpins

Genomic locations of miRNA hairpins were extracted from miRBase and converted to the hg18 assembly using the liftover tool. Genomic coordinates of small RNA sequences (25 to 36 nt) from HeLa and HepG2 cells were mapped to miRNA hairpin locations.

### EvoFold Analysis

Lists of long CDS and 5′UTR hairpins and their location in the human genome (mapping based on May 2004 release) were downloaded from the EvoFold database (available online at:http://www.cbse.ucsc.edu/~jsp/EvoFold/) [16]. The genomic coordinates were converted to the mouse genome (version mm8) using the LiftOver tool at the UCSC genome browser. Predicted hairpins were then mapped to mouse exons from UCSC known genes and Ensemble data sets and matched to the corresponding Affy IDs. Small RNAs were mapped to the hairpins using genomic coordinates using the same approach used when mapping small RNAs to exons.

For analysis of data from HeLa and HepG2 cells, EvoFold UTR and CDS hairpin coordinates were converted to this version using the Liftover tool at the UCSC genome browser [32]. Small RNAs were directly mapped to the hairpins as described earlier.

## Supporting Information

Table S1 (0.66 MB TIF)

Table S2 (3.62 MB TIF)

Table S3 (1.56 MB TIF)

Figure S1 Distribution of small RNA reads from Dgcr8 KO and WT libraries across the Hn1 exon. Small RNA locations are presented as in Figure 2 (WT reads = black bars, Dgcr8 KO reads = grey bars). Genomic coordinates are based on UCSC Known Genes annotations (mm8).
(0.66 MB TIF)

Figure S2 Distribution of small RNA reads from Dgcr8 KO and WT libraries across the Atbf1 exon, presented as in Figure S1.
(0.31 MB TIF)

Figure S3 Distribution of small RNA reads from Dgcr8 KO and WT libraries across the Adam23 exon, presented as in Figure S1.
(0.26 MB TIF)

Figure S4 Distribution of small RNA reads from Dgcr8 KO and WT libraries across the Zfp462 exon, presented as in Figure S1.
(0.43 MB TIF)

Figure S5 Distribution of small RNA reads from Dgcr8 KO and WT libraries across the Arrdc3 exon, presented as in Figure S1.
(0.45 MB TIF)

Figure S6 Distribution of small RNA reads from Dgcr8 KO and WT libraries across the Arrdc3 exon, presented as in Figure S1.
(0.55 MB TIF)

Figure S7 Distribution of 25 to 36 nt sequence reads from HeLa and HepG2 cell <200nt libraries across pre-miRNA hairpins. Locations are presented as in Figure 2. Genomic coordinates are based on mirbase annotations.
(0.71 MB TIF)
