# Supplementary figures and images for: Genomic Analysis Suggests that mRNA Destabilization by the Microprocessor Is Specialized for the Auto-Regulation of Dgcr8
Source: PLoS One. 2009 Sep 11;4(9):e6971. doi: 10.1371/journal.pone.0006971 (PMC2736397; doi:10.1371/journal.pone.0006971)

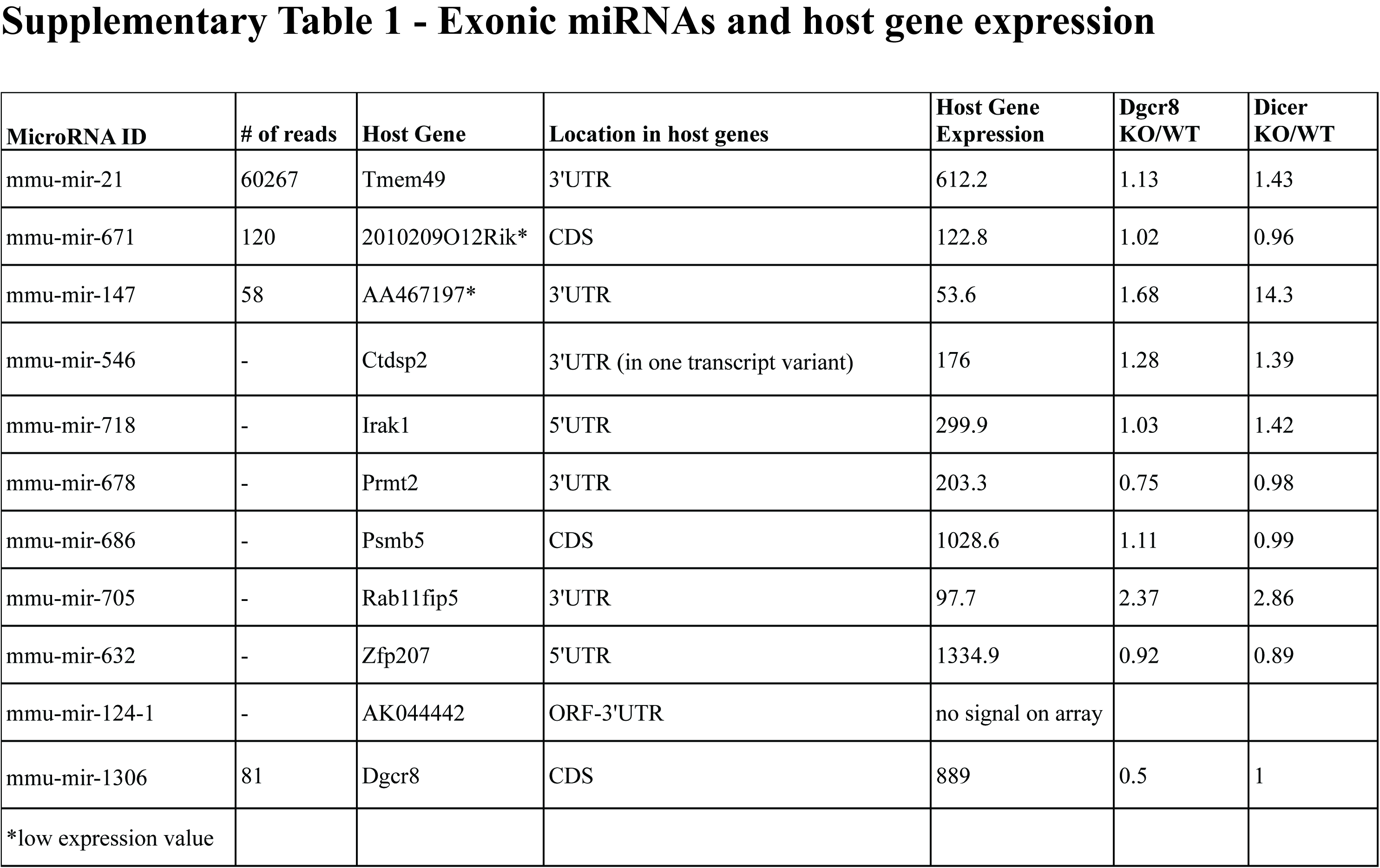

Supplement: Table S1 — (0.66 MB TIF) [file pone.0006971.s001.tif]

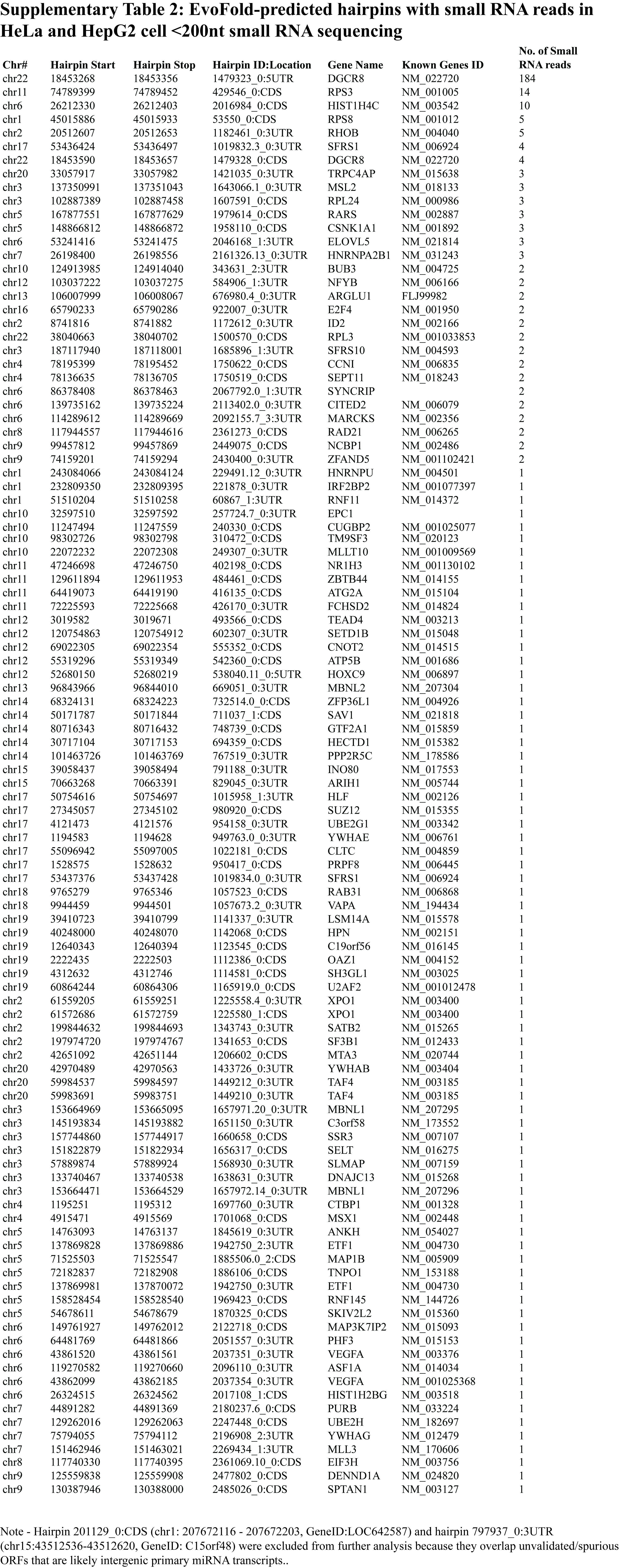

Supplement: Table S2 — (3.62 MB TIF) [file pone.0006971.s002.tif]

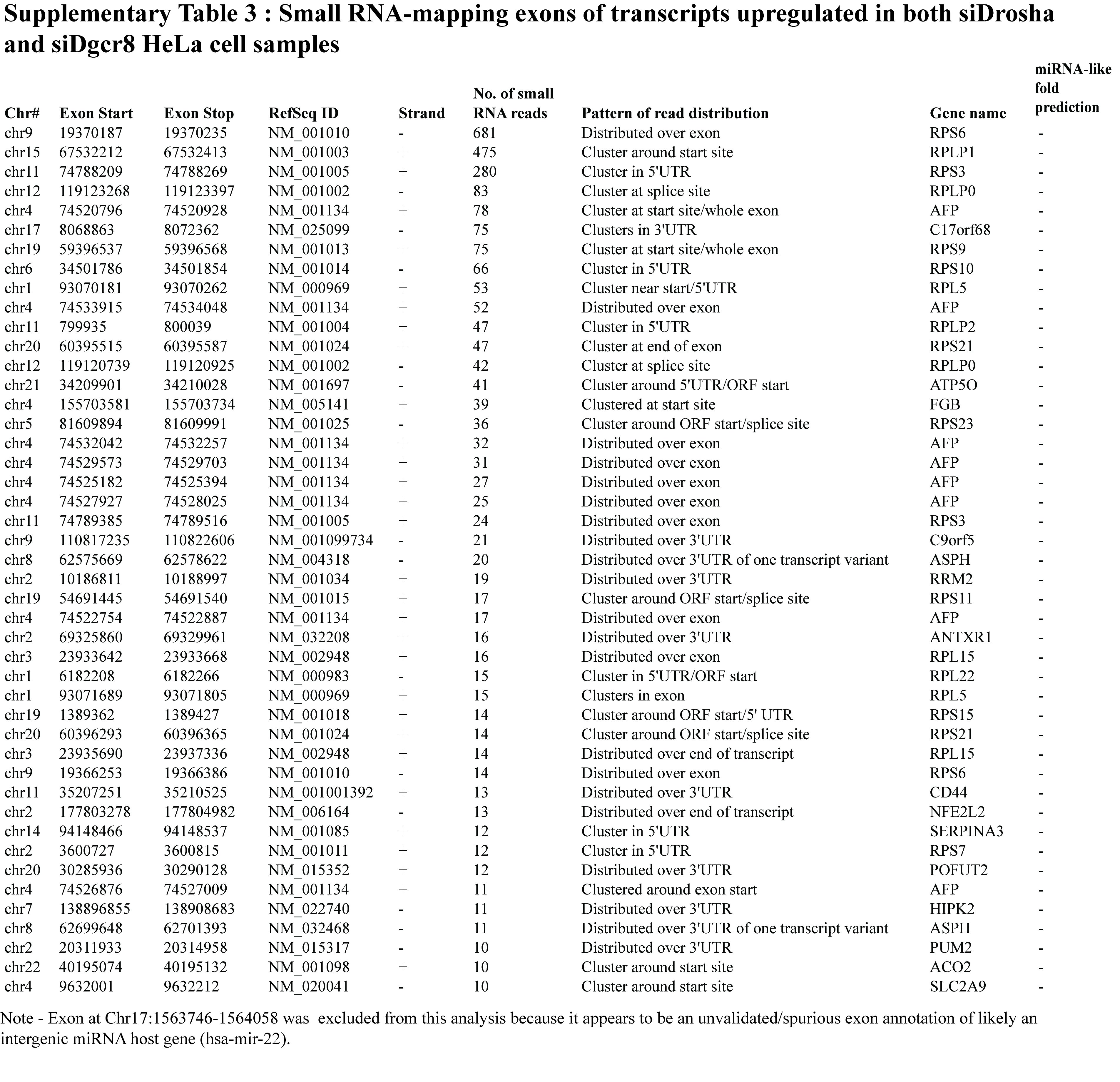

Supplement: Table S3 — (1.56 MB TIF) [file pone.0006971.s003.tif]

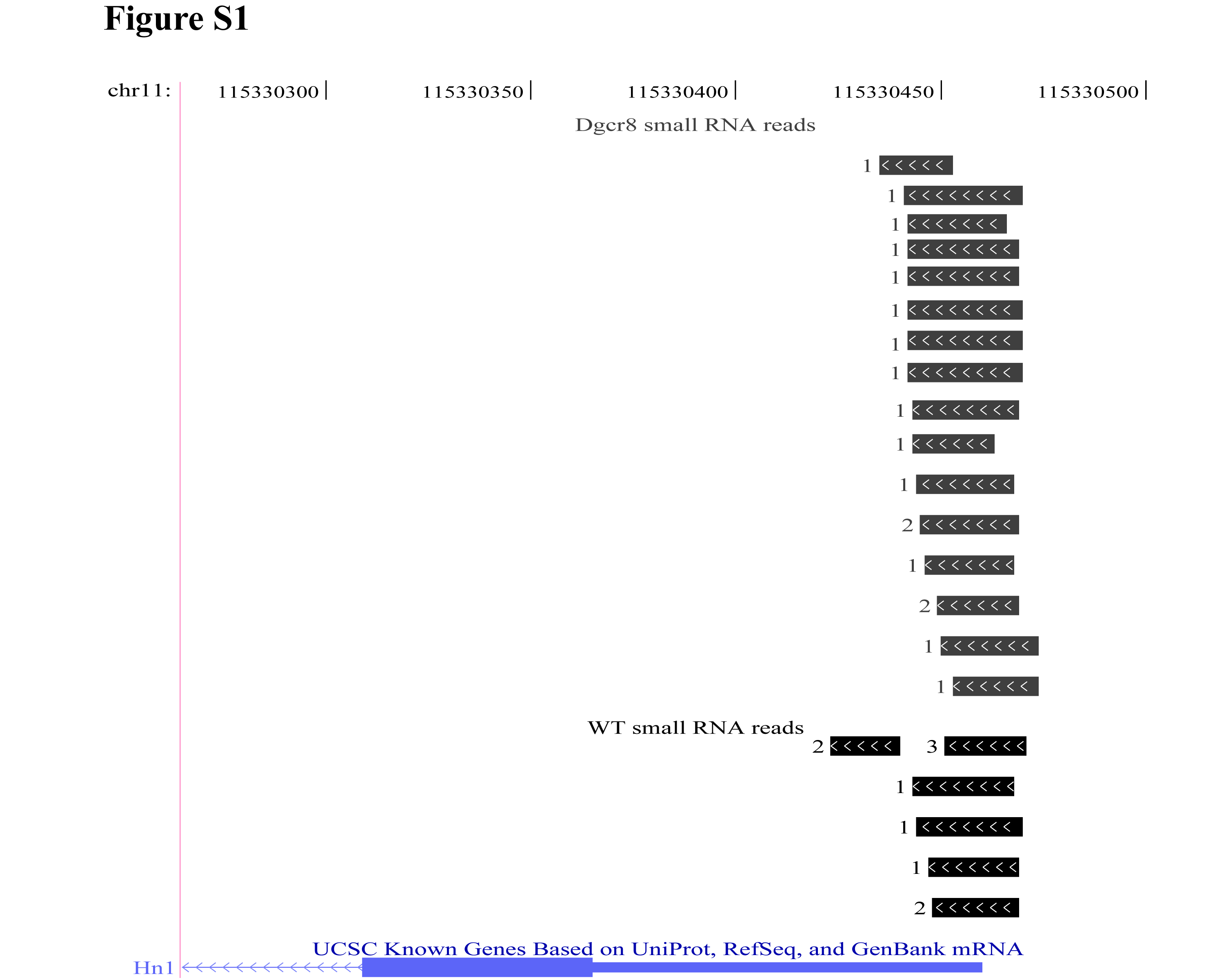

Supplement: Figure S1 — Distribution of small RNA reads from Dgcr8 KO and WT libraries across the Hn1 exon. Small RNA locations are presented as in Figure 2 (WT reads = black bars, Dgcr8 KO reads = grey bars). Genomic coordinates are based on UCSC Known Genes annotations (mm8). (0.66 MB TIF) [file pone.0006971.s004.tif]

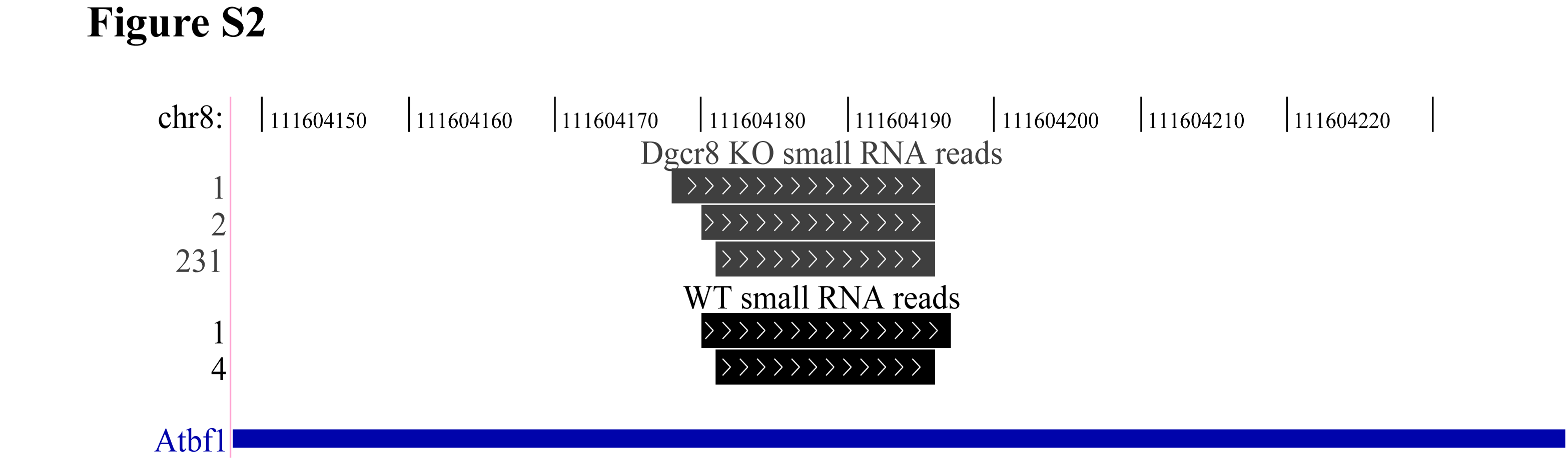

Supplement: Figure S2 — Distribution of small RNA reads from Dgcr8 KO and WT libraries across the Atbf1 exon, presented as in Figure S1. (0.31 MB TIF) [file pone.0006971.s005.tif]

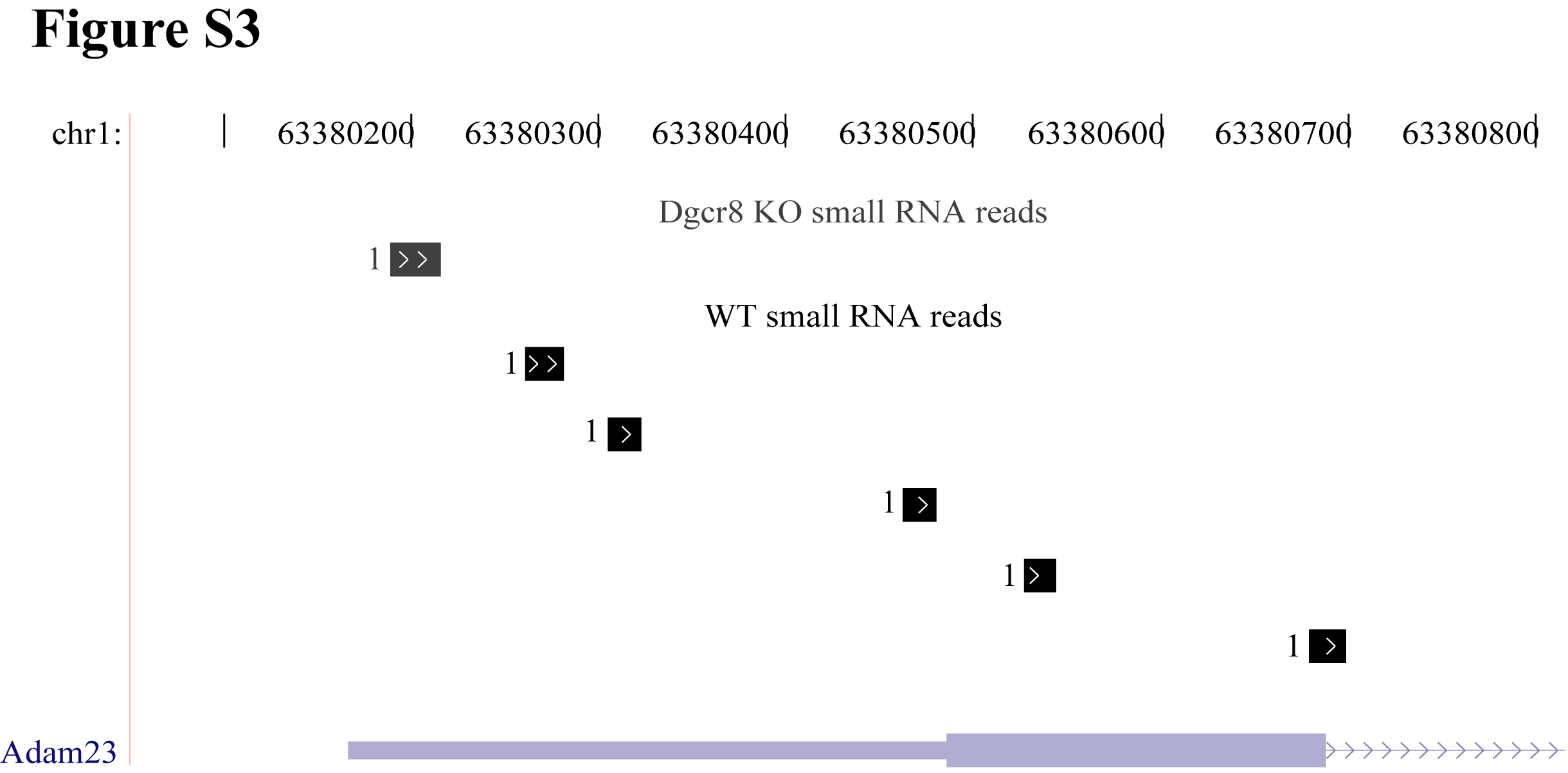

Supplement: Figure S3 — Distribution of small RNA reads from Dgcr8 KO and WT libraries across the Adam23 exon, presented as in Figure S1. (0.26 MB TIF) [file pone.0006971.s006.tif]

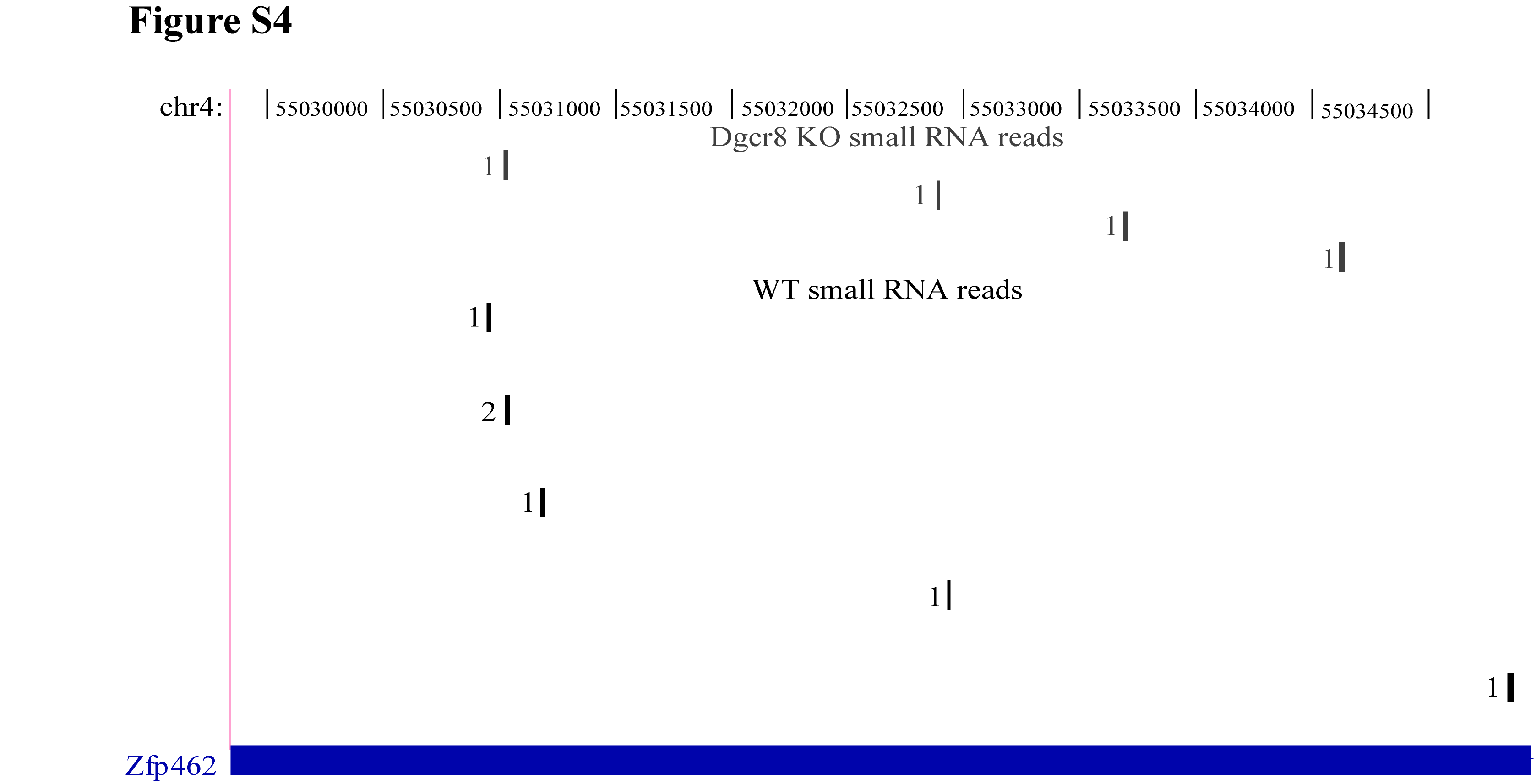

Supplement: Figure S4 — Distribution of small RNA reads from Dgcr8 KO and WT libraries across the Zfp462 exon, presented as in Figure S1. (0.43 MB TIF) [file pone.0006971.s007.tif]

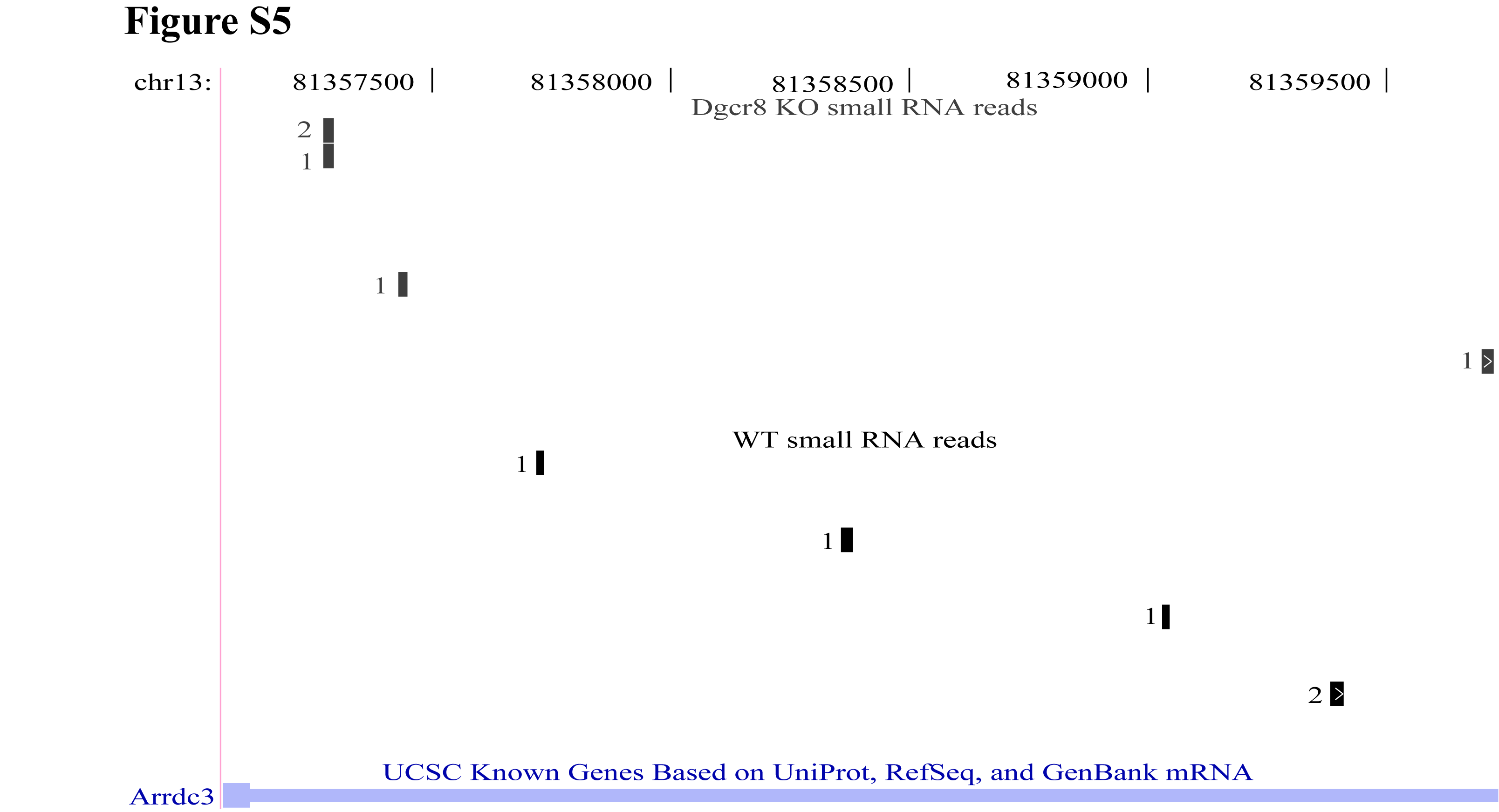

Supplement: Figure S5 — Distribution of small RNA reads from Dgcr8 KO and WT libraries across the Arrdc3 exon, presented as in Figure S1. (0.45 MB TIF) [file pone.0006971.s008.tif]

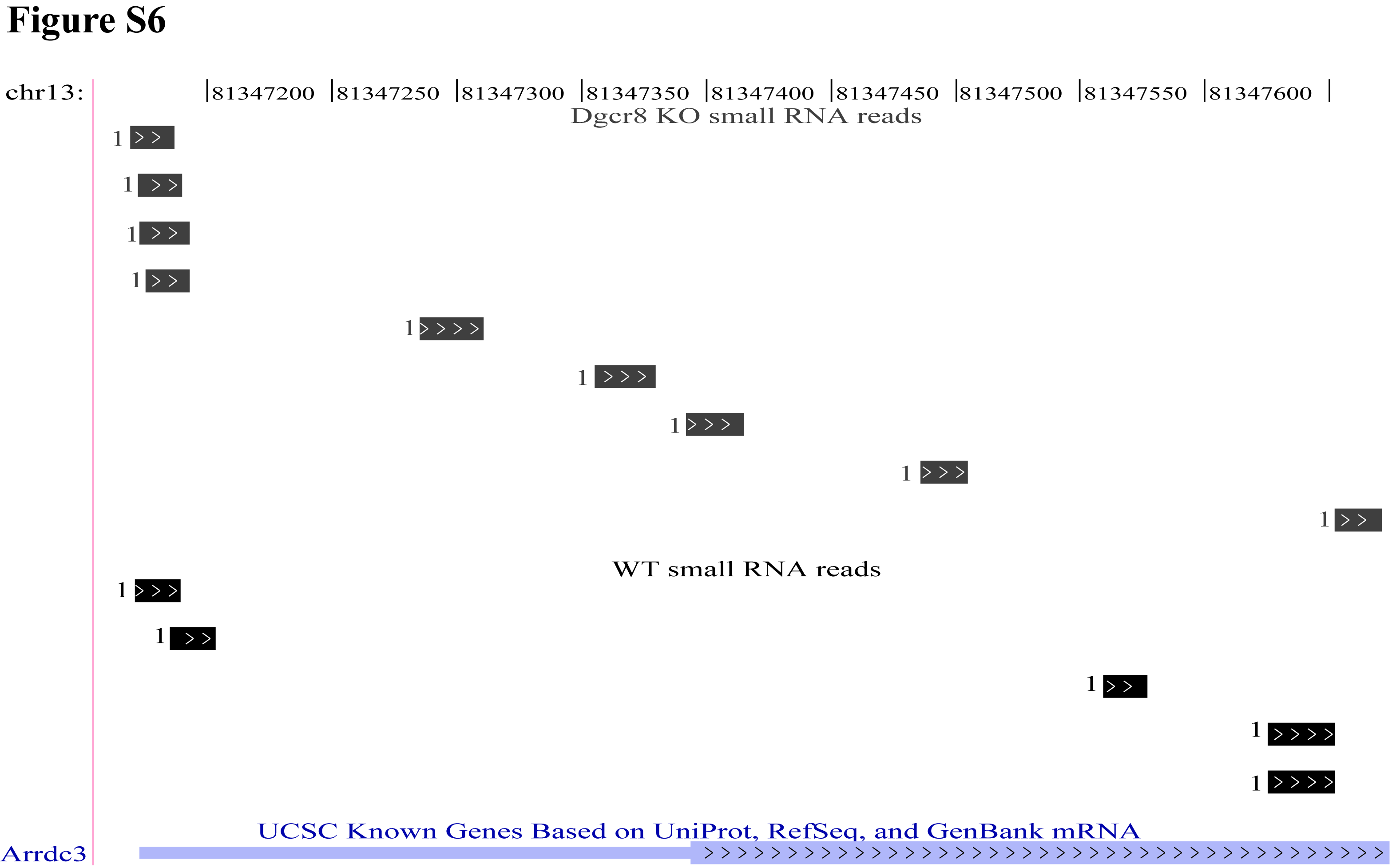

Supplement: Figure S6 — Distribution of small RNA reads from Dgcr8 KO and WT libraries across the Arrdc3 exon, presented as in Figure S1. (0.55 MB TIF) [file pone.0006971.s009.tif]

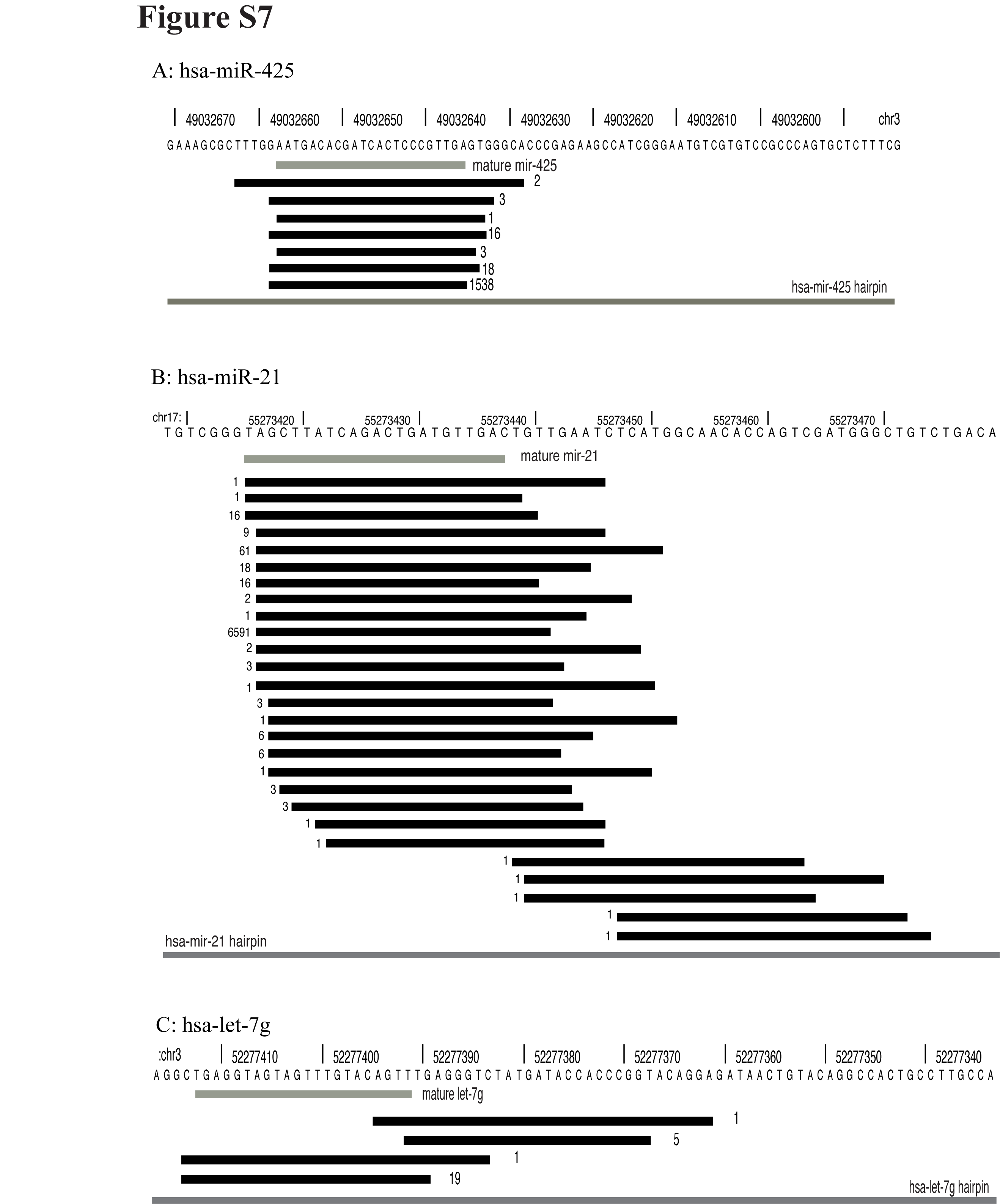

Supplement: Figure S7 — Distribution of 25 to 36 nt sequence reads from HeLa and HepG2 cell <200nt libraries across pre-miRNA hairpins. Locations are presented as in Figure 2. Genomic coordinates are based on mirbase annotations. (0.71 MB TIF) [file pone.0006971.s010.tif]
